# Binding of RAGE and RIPK1 induces cognitive deficits in chronic hyperglycemia‐derived neuroinflammation

**DOI:** 10.1111/cns.14449

**Published:** 2023-09-04

**Authors:** Xiaoyan Zhou, Yandong Zhu, Lin Gao, Yan Li, Hui Li, Chengyu Huang, Yan Liu, Ankang Hu, Changjiang Ying, Yuanjian Song

**Affiliations:** ^1^ Xuzhou Engineering Research Center of Medical Genetics and Transformation, Department of Genetics Xuzhou Medical University Xuzhou Jiangsu China; ^2^ The Graduate School Xuzhou Medical University Xuzhou Jiangsu China; ^3^ Lab Animal Center Xuzhou Medical University Xuzhou China; ^4^ Department of Endocrinology Affiliated Hospital of Xuzhou Medical University Xuzhou Jiangsu China

**Keywords:** cognitive deficits, diabetes, Neuroinflammation, RAGE, RIPK1

## Abstract

**Aims:**

Chronic hyperglycemia‐induced inflammation of the hippocampus is an important cause of cognitive deficits in diabetic patients. The receptor for advanced glycation end products (RAGE), which is widely expressed in the hippocampus, is a crucial factor in this inflammation and the associated cognitive deficits. We aimed to reveal the underlying mechanism by which RAGE regulates neuroinflammation in the pathogenesis of diabetes‐induced cognitive impairment.

**Methods:**

We used db/db mice as a model for type 2 diabetes to investigate whether receptor‐interacting serine/threonine protein kinase 1 (RIPK1), which is expressed in microglia in the hippocampal region, is a key protein partner for RAGE. GST pull‐down assays and AutoDock Vina simulations were performed to identify the key structural domain in RAGE that binds to RIPK1. Western blotting, co‐immunoprecipitation (Co‐IP), and immunofluorescence (IF) were used to detect the levels of key proteins or interaction between RAGE and RIPK1. Cognitive deficits in the mice were assessed with the Morris water maze (MWM) and new object recognition (NOR) and fear‐conditioning tests.

**Results:**

RAGE binds directly to RIPK1 via the amino acid sequence (AAs) 362–367, thereby upregulating phosphorylation of RIPK1, which results in activation of the NLRP3 inflammasome in microglia and ultimately leads to cognitive impairments in db/db mice. We mutated RAGE AAs 362–367 to reverse neuroinflammation in the hippocampus and improve cognitive function, suggesting that RAGE AAs 362–367 is a key structural domain that binds directly to RIPK1. These results also indicate that hyperglycemia‐induced inflammation in the hippocampus is dependent on direct binding of RAGE and RIPK1.

**Conclusion:**

Direct interaction of RAGE and RIPK1 via AAs 362–367 is an important mechanism for enhanced neuroinflammation in the hyperglycemic environment and is a key node in the development of cognitive deficits in diabetes.

## Graphical abstract

Proposed mechanism underlying RAGE regulation of cognitive deficits. Direct binding of RAGE AAs 362–367 to RIPK1 is responsible for activation of RIPK1 and the NLRP3 inflammasome, leading to subsequent cognitive deficits.

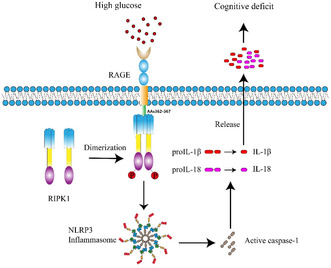

## 1 INTRODUCTION

Type 2 diabetes mellitus (T2DM) is a chronic metabolic disease prevalent across the world. T2DM produces chronic low‐grade inflammation in the brain and leads to severe co‐morbidities, such as neuroinflammation and neurodegenerative disease.^1^, ^2^ The persistent inflammatory response contributes to microglial activation and cognitive impairments; in both patients and rodents, diabetes results in enhanced risk of progressive dementia.^3^ The hippocampus, located in the medial temporal lobe, is a critical brain region for learning and memory and exhibits early damage during the progression of diabetes.^4^ Furthermore, the hippocampus is readily impacted by inflammatory factors associated with microglial activation.^5^ Thus, the inflammatory response in the hippocampus is thought to be involved in the development and progression of cognitive deficits induced by diabetes.^6^, ^7^

The receptor for advanced glycation end products (RAGE) is a prominent mediator of inflammation in diabetes and is upregulated in the hippocampus.^8^ RAGE is a pattern‐recognition receptor and is constitutively expressed in neurons, astrocytes, and microglia.^9^ It is composed of three extracellular immunoglobulin domains, a transmembrane helix, and a short 42‐amino‐acid cytoplasmic domain.^10^ Multiple extracellular ligands are recognized by RAGE, which then activates intracellular signaling pathways in the cytoplasmic domain, ultimately leading to cellular damage.^10^ Many signaling molecules are involved in the RAGE modulation of inflammatory responses and microglial activation.^11^, ^12^ Inhibition of RAGE attenuates microglial activation and ameliorates behavioral deficits in a mouse model of diabetes and neurodegenerative disease.^13^, ^14^ Together, these findings emphasize the important role of RAGE in neuroinflammation‐induced cognitive impairment. Nevertheless, the underlying molecular mechanisms responsible for RAGE regulation of microglial activation in a high‐glucose environment require further elucidation.

Receptor‐interacting kinase 1 (RIPK1) is a serine/threonine kinase that regulates homeostasis at the cellular and tissue levels by promoting the release of inflammatory factors through integration of inflammatory signaling pathways.^15^ In the central nervous system (CNS), RIPK1 is highly expressed in microglia after inflammatory stimulation.^16^, ^17^ RIPK1 is composed of an N‐terminal serine/threonine kinase domain, an intermediate domain, and a C‐terminal death domain (DD).^18^ Enhanced activity of RIPK1 in microglia is closely associated with the pathological processes of several human inflammatory and degenerative diseases.^15^, ^19^, ^20^ Emerging evidence suggests that RIPK1 is involved in mediating the activation of nucleotide‐binding oligomerization domain (NOD), leucine‐rich repeats, and the pyrin domain‐containing protein 3 (NLRP3) inflammasome to promote proinflammatory cytokine and chemokine production and induce a detrimental neuroinflammatory environment.^20^, ^21^ Inactivation of RIPK1 kinase by Nec‐1 s or mutation can protect against the RIPK1‐induced neuroinflammatory response and improve cognitive deficits in animal models of neurodegeneration.^19^ It remains unclear whether RIPK1 regulatory mechanisms are involved in mediating neuroinflammation in diabetes.

We propose that increased neuroinflammation in chronic hyperglycemia may be due to overexpression of RAGE, subsequent RIPK1 phosphorylation in microglia, and activation of the associated signal transduction pathway and that these changes ultimately lead to cognitive deficits. Here, we show that RAGE binds to RIPK1 in hippocampal microglia under chronic diabetes conditions. Specifically, we demonstrate the key structural basis by which RAGE combines directly with RIPK1. In addition, we show that the RIPK1‐dependent NLRP3 inflammasome is activated in microglia during diabetes. Finally, mutating RAGE attenuates neuroinflammation and ameliorates cognitive dysfunction in db/db mice by blocking the RAGE–RIPK1 interaction and thus dampening the inflammasome.

## 2 MATERIALS AND METHODS

### 2.1 Antibodies and reagents

All chemicals, antibodies, recombinant proteins, critical commercial assays, cell lines, experimental mice, oligonucleotides, and recombinant DNA are listed in Supplementary Table S1.

### 2.2 BV2 cell line: experimental design

Cells from the BV2 microglial cell line were cultured in Dulbecco's modified Eagle's medium (DMEM) with 10% fetal bovine serum (FBS) and 1% penicillin/streptomycin. Cells were kept at 37°C with 20% O_2_ and 5% CO_2_, and 0.05% trypsin/EDTA was applied for cell passaging. Cells were grown in serum‐free medium for 12 h and were randomly assigned to different groups: a normal group (NG; 25 mM glucose), a high‐glucose group (HG; 60 mM glucose), a RAGE‐inhibitor group (FPS; same as the high‐glucose group but treated with 2.5 μM FPS‐ZM1 dissolved in DMSO), a solvent control group (DMSO; same as the high‐glucose group but treated with DMSO), and a mannitol control group (MG; 25 mM glucose plus 35 mM mannitol as an isosmotic pressure control). After 24 h, cells were harvested and used in the experiments.

For cell transfection, BV2 microglia were seeded at 2 × 10^4^ cells/well in 6‐well plates. After reaching 60% confluence, cells were transferred to serum‐free DMEM for synchronization for 12 h, and RIPK1 or/and RAGE plasmids were transfected using 50 nmol Lipofectamine 3000 reagent for 48 h, according to the manufacturer's protocol. Then, cells were collected for further study.

### 2.3 Mouse experiments: experimental design

Male db/db mice (BKS.Cg‐m+/+ Leprdb/J) and their age‐ and gender‐matched normoglycemic heterozygous littermate db/m (BKS.Cg‐m+/+ Leprdb/+/J) controls were purchased from Jackson Laboratory.^22^, ^23^ Mice were kept in specific pathogen‐free conditions and housed in cages with controlled temperature (20–25°C) and light (12 h light/12 h dark). Mice were housed 4–5 per cage and had free access to water and food. The animals were treated humanely, and all efforts were made to minimize the animals' suffering and the number of mice used in the study.

Male db/db mice (7–8 weeks old) were randomly divided into four groups: a db/db group (db/db; mice did not receive any treatment), an FPS‐ZM1 group (FPS; db/db mice received 3.33 mL/kg/d, i.p. of FPS‐ZM1 (1.0 mg/kg) dissolved in corn oil), a corn oil group (Oil; db/db mice received 3.33 mL/kg/d, i.p., of corn oil), and an age‐matched db/m control group (db/m). After 8 weeks of treatment administration, mice were sacrificed for further study.

In a different experiment, 7–8‐week‐old male db/db mice received bilateral micro‐injections of lentivirus (LV)‐RAGE‐shRNA (1 μL) in the hippocampus to knock down RAGE (db/db + RAGE‐KD). Mice were anesthetized with 1.5% pentobarbital (0.6 mL/g) and then fixed in a stereotaxic frame. The hippocampal CA1 subregion was targeted bilaterally at AP –2.0 mm, ML ± 1.7 mm, and DV –2.2 mm. The injection velocity was 0.25 μL/min, and the micro‐injector was kept in place for 5 min after the injection. At 9–10 weeks of age, adeno‐associated virus (AAV, 1 μL) engineered to overexpress GFP‐tagged wild‐type RAGE (db/db + RAGE‐KD + Wt), mutant RAGE (db/db + RAGE‐KD + Mut), or a nonsense control (db/db + RAGE‐KD + NC) under the microglial‐specific CD68 promoter was bilaterally micro‐injected into the hippocampus using the same method. After waiting 8 weeks for recovery and expression, further experiments were performed.

### 2.4 Protein collection and extraction

BV2 cells were washed three times with PBS and then centrifuged at 800 *g* for 10 min to collect the protein precipitate. For animal protein collection, mice were anesthetized, and then, the hippocampus was dissected carefully on ice and immediately frozen in liquid nitrogen and stored at −80°C. Proteins were extracted with cytoplasmic protein extraction kits. Briefly, three volumes of cold homogenization buffer A containing phosphatase inhibitor cocktails and phenylmethanesulfonyl fluoride (PMSF) were added to the protein sample, the mixture was centrifuged at 1200 *g* for 5 min, and the supernatants were taken as the cytosolic fraction. A BCA Protein Assay Kit was used for quantification and protein concentrations were determined by the Lowry method.

### 2.5 Western blotting

For each sample, 30 μL of proteins mixed with 4× loading buffer was separated on SDS‐PAGE gels and then transferred to nitrocellulose membrane (Amersham Biosciences), blocked with 3% bovine serum albumin for 1 h at room temperature. Membranes were incubated first with the appropriate primary antibodies overnight at 4°C and then washed with TBS‐T for 5 min three times the next day. Then, membranes were incubated with the appropriate secondary antibodies for 2 h at room temperature. The signals from the immunoreactive bands were scanned with an Odyssey Laser imaging scanner (Olympus FV10i) and analyzed in ImageJ.

### 2.6 Co‐immunoprecipitation

200 μg of proteins were diluted with 400 μL immunoprecipitation buffer. Co‐immunoprecipitation was performed by incubating the indicated antibodies or control IgG overnight at 4°C followed by adding protein A/G‐Agarose for 2 h at 4°C. Then, the mixture was washed five times by centrifugation at 1000 *g* for 2 min at 4°C with immunoprecipitation buffer. The supernatants were eluted by addition of sample buffer and then subjected to western blotting.

### 2.7 Liquid chromatograph–mass spectrometer analysis

The hippocampus of db/db and db/m mice was carefully dissected out on ice, and then, proteins were extracted with immunoprecipitation buffer. Afterward, 400 μg of proteins were incubated with RAGE primary antibody for 16 h; protein A/G‐Agarose was added to the mixture for 2 h at 4°C the next day. After washing with immunoprecipitation buffer, protein supernatants were electrophoresed in SDS‐PAGE gels. The gels were tested by BGI Genomics, and the RAGE protein abundance was calculated.

### 2.8 Immunofluorescence

To assess co‐localization of RAGE and RIPK1, BV2 cells were fixed with 4% paraformaldehyde and permeabilized with PBS containing 0.3% Triton X‐100 for 30 min. Cells were incubated with RAGE (1:300) and RIPK1 (1:200) primary antibodies overnight at 4°C followed by blocking with 1% goat serum for 1 h. After rinsing three times for 3 min with 1% PBS, cells were incubated with Alexa Fluor 488‐conjugated rabbit or Alexa Fluor 594‐conjugated mouse secondary antibodies (1:1000) for 2 h. Cells were washed three time for 8 min and mounted with DAPI. Images were captured with a confocal microscope (Zeiss LSM710), and the overlap value (the percentage of co‐localization of red fluorescence and green fluorescence) was analyzed by the Zeiss system software.

Mice in different groups were perfused with 0.9% saline, followed by 4% paraformaldehyde. Brains were removed and postfixed for 24 h, and then, gradient was dehydrated with 15% and 30% sucrose in 0.1 M PBS. Subsequently, the hippocampus was cut longitudinally into 20 μm sections using a cryostat and mounted on slides. The frozen hippocampus sections were permeabilized with 0.3% Triton X‐100 in PBS for 30 min and then blocked with non‐specific IgG for 1 h at room temperature. Next, sections were incubated with the anti‐iba‐1 primary antibody (1:200) at 4°C overnight. After washing three times, sections were incubated with the fluorescence‐labeled secondary antibody for 2 h at room temperature in the dark then washed three times for 8 min followed by mounting with DAPI. DAPI and Iba‐1 positive cells were considered to be activated microglia. The number of activated microglia per 1‐mm length of the hippocampal CA1 subregion was then counted.

### 2.9 Docking methods

The C‐terminal of RAGE (*Mus musculus*, residues 362–402) was obtained from the Protein Data Bank database (https://www.rcsb.org/structure/2LMB), and the death domain (DD) of the RIPK1 model was built with Swiss‐Model (https://swissmodel.expasy.org/). Docking experiments were conducted by AutoDock Vina (version 1.1.2).^24^ Amino acids in the C‐terminal of RAGE were set as flexible residues and, except for peptide bonds, all bonds could rotate. A genetic algorithm was used to minimize system energy and prepare docking conformations using the default settings, and about 50 possible substrate conformations were established by docking. The conformations with the highest docking score are presented and were used in subsequent analyses. All model figures representing protein interactions were created in PyMOL (version 0.99).

### 2.10 GST pull‐down assay

The GST‐tagged vector pGEX‐4 T‐1 was used to express RAGE and two RAGE mutations (amino acid sequence (AAs) 362–367 and AAs 383–385). GST and GST‐tagged wild‐type/mutant RAGE were translated and over‐expressed in BL21 cells, followed by protein purification according to a GST protein interaction pull‐down kit. His‐tagged RIPK1 in pIRES2 was transfected into BV2 cells, and proteins were lysed in homogenization buffer after overexpression. The GST pull‐down assay was executed according to the manufacturer's instructions. Briefly, 50 μL of agarose resin was pre‐equilibrated in TBS containing the pull‐down lysis buffer. GST‐tagged RAGE (200 μL) was incubated with the resin at 4°C for 6–8 h on a rotating platform. After washing five times, the RIPK1 reaction products were co‐incubated with GST‐tagged protein for 2 h. Finally, the protein interaction was detected and analyzed by Western blotting.

### 2.11 Intrahippocampal injections

Mice were anesthetized with 1.5% pentobarbital (0.6 mL/g) and then fixed in a stereotaxic frame. 2 μL LV‐RAGE‐shRNA or LV‐NC were injected into the hippocampal CA1 subregion bilaterally at a location of AP –2.0 mm, ML ± 1.7 mm, and DV –2.2 mm. The injection velocity was 0.25 μL/min, and the micro‐injector was kept in place for 5 min after the injection. Two weeks later, RAGE knockdown was confirmed and AAV‐GFP‐RAGE‐Wt or AAV‐GFP‐RAGE‐Mut under the microglia‐specific CD68 promoter was injected bilaterally following the same procedures.

### 2.12 Behavioral studies

Behavioral testing began when mice were 17–18 weeks old. First, the Morris water maze (MWM) test was performed for 6 consecutive days, then the fear‐conditioning test was conducted for 2 consecutive days, and then, the novel object recognition (NOR) test was performed for 3 consecutive days. There was an interval of 24 h between each experiment. Behavioral tests were recorded with a CCD (charge‐coupled device) ANY‐maze video tracking system (Stoelting 7.1.5) and analyzed with the ANY‐maze system‐specific software.

#### 2.12.1 Morris water maze

The MWM was a round stainless steel pool with a diameter of 120 cm, filled with opacified (non‐toxic white paint) water, and located in a dimly lit room. The height of the water was 30 cm, and the temperature was 22–26°C. The pool was divided into four symmetrical quadrants with four visual cues and an invisible escape platform hidden 1 cm beneath the surface of the water in a fixed quadrant. The test was performed as a series of 4 trials per day for 5 days followed by a probe trial on the sixth day. On the five training days, each mouse was placed in the water facing the wall and allowed to search for the hidden platform for 60 s. When the mouse arrived at the platform, it was permitted to stay on the platform for 10 s; if the mouse was unable to find the platform in the allotted time, it was guided manually to the platform and given 10 s to remember associated spatial cues. Each mouse was tested 4 times per day with trials 1 h apart, starting from a different quadrant each time (in a randomized order). The probe trial was conducted on day 6: the hidden platform was removed and mice swam freely for 60 s. The ratio of time and swimming distance spent in the vicinity of the platform and the number of times the mouse crossed the prior position of the platform were recorded and analyzed.

#### 2.12.2 Fear‐conditioning

The fear‐conditioning test was performed in a plexiglas training chamber with a stainless‐steel grid floor for shock delivery. The mouse was placed in the chamber and allowed to observe the environment for 3 min; then, three 30‐s tone stimuli (2000 Hz, 70 db) were presented, accompanied by a shock (0.7 mA) during the last 2 s of the tone. After three tone–shock pairings with a 1‐min interval, the mouse was returned to the home cage and the training chamber was cleaned with 95% ethyl alcohol before the next animal. On the following day, the mouse was returned to the training chamber for 8 min with delivery of neither tone nor foot shock, and the ratio of freezing time to the total 8 min was assessed as a measure of contextual fear conditioning. After 2 h, the mouse was placed in a novel chamber for a 3‐min adaptation period then the 2000 Hz, 70 db tone was presented continuously for 30‐s in the absence of shocks. The ratio of freezing time to the total 3 min was used as an indicator of cued fear conditioning.

#### 2.12.3 New object recognition

This test was performed over two consecutive days. Initially, on the first day (T0), the experimental mouse was placed individually in a 40 × 40 cm open arena for 5 min. The next day, the mouse was placed into the same arena for 5 min but with two objects of the same shape and color, and the amount of time the mouse spent exploring the objects was recorded. 1 h later, one of the objects was changed for a new one that differed both in shape and color, and the time the mouse spent exploring each object over 5 min was recorded and the difference represented as a discrimination index (DI). Because mice have an innate preference for novelty, mice without cognitive impairment will recognize the familiar object and will spend most of the time exploring the novel object. The DI was calculated as the time spent with each object (novel−familiar/total object exploration), so a positive number indicates a preference for the novel object and a negative number indicates a preference for the familiar object.

### 2.13 Statistical analysis

All data were analyzed in GraphPad Prism 7.0. We first checked the data for normality (Shapiro–Wilk test) and equal variance (*F*‐test). When the data met the assumptions for parametric tests, a Student's t‐test was used to compare two groups and a one‐way analysis of variance (ANOVA) followed by Tukey post hoc tests was used for comparisons among multiple groups. Data from the MWM training sessions were analyzed with a two‐way ANOVA followed by Tukey multiple comparisons tests. *p* < 0.05 was considered statistically significant. All experimental results are shown as the mean ± standard deviation. Sample sizes were based on those used in previous studies in this field. Animals were randomly assigned to experimental groups. The investigator was blinded to the group allocations during the experiments and when assessing the outcomes.

## 3 RESULTS

### 3.1 RAGE interacts with RIPK1 and enhances RIPK1 phosphorylation under hyperglycemic conditions

**FIGURE 1:**
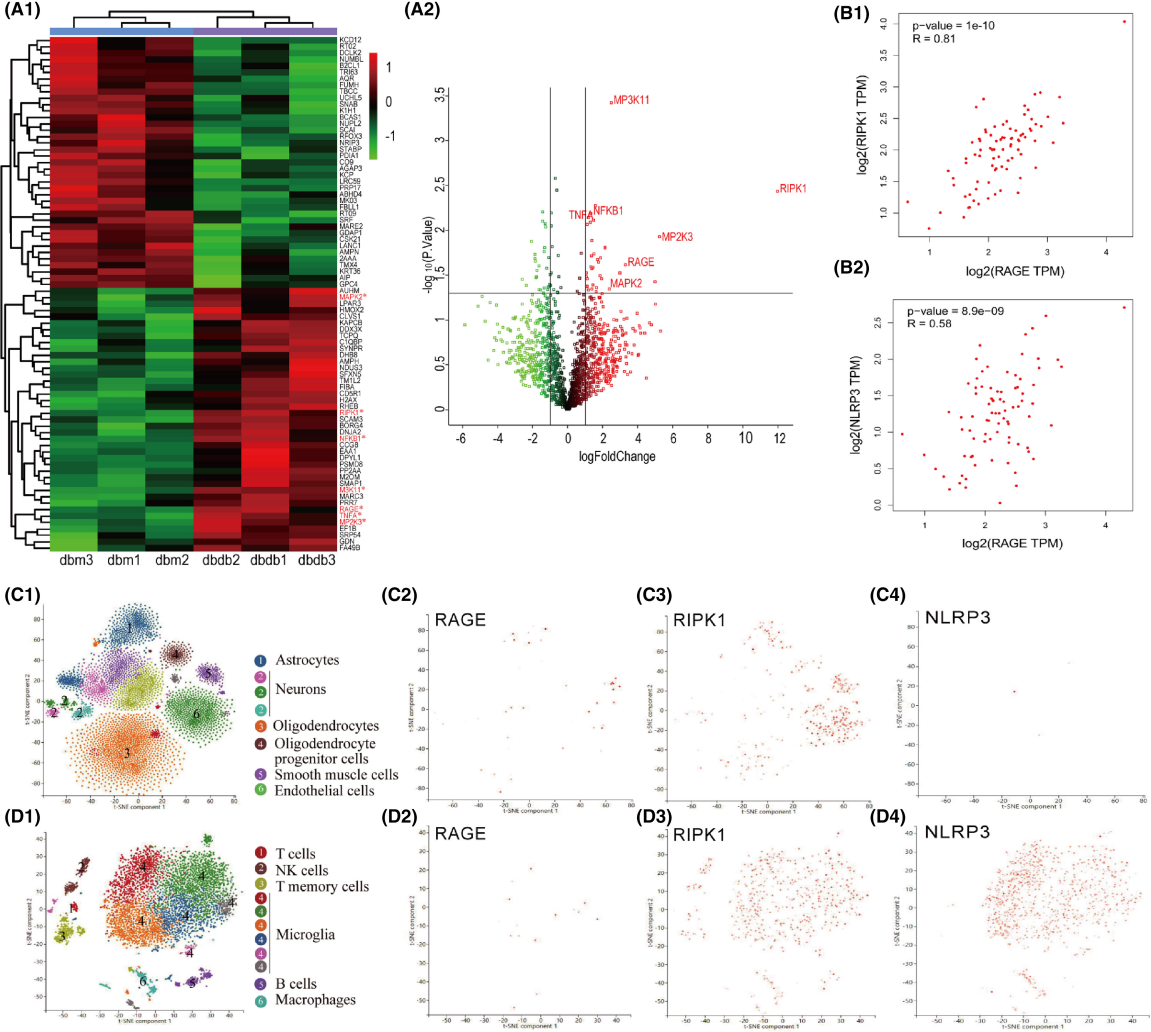
RIPK1 interacts with RAGE in microglia under hyperglycemic conditions. (A1) Heatmap of RAGE‐related proteins quantified from the hippocampus of db/m and db/db mice. Red indicates an increase in the protein, and green indicates a decrease in the protein. (A2) Volcano plot from the mass spectrometry results showing differentially expressed proteins. The abscissa is the logarithm of the fold change in differential protein expression in the comparison group. Each point represents a specific protein. Upregulated proteins are labeled in red, and downregulated proteins are labeled in green. Proteins that were not significantly differentially expressed are labeled in black. (B1 and B2) The correlations between RAGE and RIPK1 (*R* = 0.81), and between RAGE and downstream NLRP3 (*R* = 0.58) were analyzed with GEPIA. (C1–D4): PanglaoDB was used for single‐cell RNA sequencing localization analysis of RAGE, RIPK1, and NLRP3 in the brain. RAGE was expressed in a wide range of cell types in the brain, whereas RIPK1 and NLRP3 were mostly in the microglia cluster.

Given the crucial role of RAGE in hyperglycemia‐induced neuroinflammation, we investigated RAGE‐interacting proteins in the hippocampus of db/db mice through liquid chromatograph–mass spectrometry (LC–MS) analysis. We found a significant increase in the abundance of proteins associated with the mitogen‐activated protein kinase (MAPK) signaling pathway in db/db mice.^25^ In particular, the abundance of RIPK1 protein, which can induce neurodegenerative disease by enhancing the inflammatory response, was significantly elevated in db/db mice (Figure 1A1,A2). Consistent with this, analysis by the “Correlation Analysis” tool of the Gene Expression Profiling Interactive Analysis (GEPIA) database showed that both RIPK1 and the downstream NLRP3 inflammasome were positively correlated with the protein expression of RAGE (*r* = 0.81 and 0.58 respectively; Figure 1B1,B2). Next, we explored the cell‐type expression of RAGE, RIPK1, and the NLRP3 inflammasome in the brain through the single‐cell database PanglaoDB and found that RAGE is ubiquitously expressed in the CNS whereas RIPK1 and NLRP3 are mostly expressed in microglia (Figure 1C1–C4,D1–D4).

Phosphorylated RIPK1 (p‐RIPK1) is the form of RIPK1 activation that plays a key role in RIPK1‐induced inflammation.^26^ Therefore, we measured the level of RIPK1 phosphorylation under high‐glucose conditions. As expected, there was an increase in RIPK1 phosphorylation (pS166) after subjecting BV2 microglia to high‐glucose (HG) conditions for 48 h, compared with normal glucose (NG) conditions (*F*(3, 12) = 86.22. HG 48 h vs NG, *p* < 0.001; Figure S1A1,A2); we used 48 h of high‐glucose for the rest of the experiments. In addition, we observed RIPK1 phosphorylation at S166 in the hippocampus of db/db mice (*t* = 5.70, db/m vs db/db, *p* < 0.001; Figure S1B1,B2). From these observations, RIPK1 activation in a high‐glucose environment may be associated with RAGE.

### 3.2 RAGE regulates RIPK1 phosphorylation through the RAGE–RIPK1 conjunction

**FIGURE 2:**
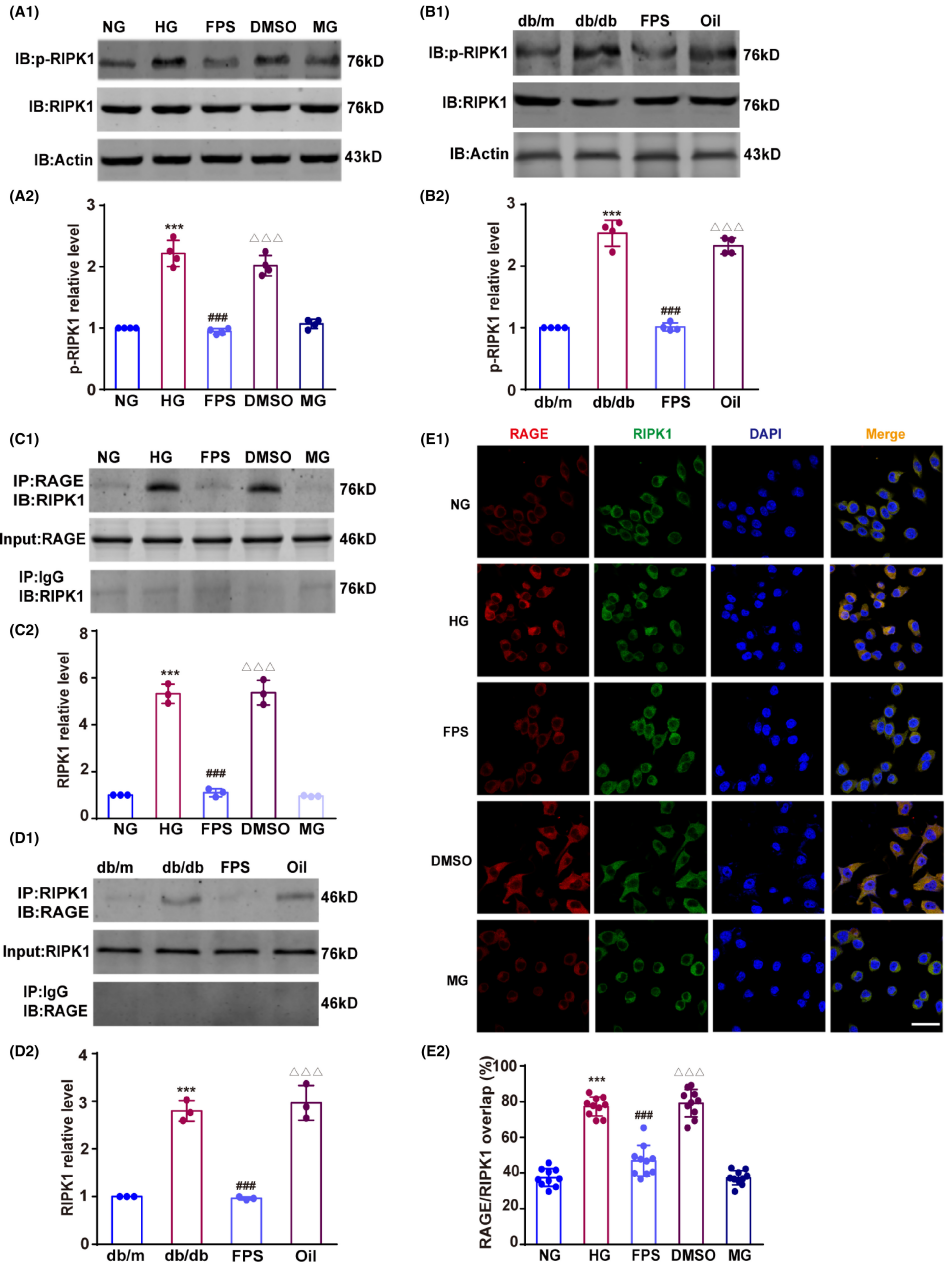
The interaction of RAGE with RIPK1 induces RIKP1 phosphorylation. NG: 25 mM glucose; HG: 60 mM glucose; FPS: high‐glucose group treated with FPS‐ZM1 dissolved in DMSO; DMSO: high‐glucose group plus DMSO; MG: 25 mM glucose plus 35 mM mannitol as an isosmotic pressure control. db/m: Age‐matched control mice; db/db: diabetic mice; FPS: db/db mice received FPS‐ZM1 1.0 mg/kg dissolved in corn oil; Oil: db/db mice treated with corn oil. (A1 and B1) Representative Western blots of p‐RIPK1 expression in BV2 cells and in hippocampus. (A2 and B2) Optical density was measured as the fold change relative to the NG group or the db/m group, *n* = 4 in each group. (C1) The combination of RAGE and RIPK1 was detected in BV2 cells. (C2) Relative intensity is displayed as the fold change relative to the NG group. *n* = 3 in each group. (D1) The interaction of RAGE and RIPK1 in hippocampus was assessed. (D2) Optical density is shown as the fold change relative to the db/m group. *n* = 3 in each group. (E1 and E2) Typical laser‐scanning confocal microscopy images and overlap values showing high‐glucose‐induced co‐localization of RAGE and RIPK1 in BV2 cells. RAGE is labeled in red, RIPK1 is labeled in green, cellular nuclei are marked in blue by DAPI, and co‐localization of RAGE and RIPK1 is indicated in yellow. Scale bar = 10 μm (400× magnification). *n* = 10 in each group. Data were presented as the mean ± S.D and were analyzed by one‐way ANOVA followed by Tukey's test. ***p < 0.001 compared with the NG group (A2, C2 and E2) or db/m group (B2 and D2). ^###^ *p* < 0.001 compared with the HG group (A2, C2 and E2) or db/db group (B2 and D2). ^△△△^ *p* < 0.001 compared with the FPS group.

Next, we validated the effect of RAGE on RIPK1 phosphorylation in high‐glucose conditions. Owing to the inherent differences between in vitro and in vivo experiments, the glucose levels are not directly comparable. The conditions of normal (25 mM) and high (60 mM) glucose used in vitro were selected on the basis of previous studies to approximate conditions in db/m and db/db mice.^27^, ^28^ In BV2 microglia, high glucose elevated the p‐RIPK1 level; the RAGE inhibitor FPS‐ZM1 blocked this increase but the FPS‐ZM1 solvent control (DMSO alone) did not (*F*(3, 12) = 94.17, HG vs NG, *p* < 0.001; FPS vs HG, *p* < 0.001; DMSO vs FPS, *p* < 0.001; Figure 2A1,A2). Because the cognitive dysfunction observed in db/db mice occurs gradually, mice were treated with FPS‐ZM1 at 7–8 weeks old and then p‐RIPK1 levels were measured at 15–18 weeks. p‐RIPK1 was increased in 15–18‐week‐old db/db mice; this increase was blocked by prior FPS‐ZM1 treatment (*F*(3, 12) = 165.30, db/db vs db/m, *p* < 0.001; FPS vs db/db, *p* < 0.001; Oil vs FPS, *p* < 0.001; Figure 2B1,B2).

Next, we tested the co‐precipitation of RAGE with RIPK1. As shown in Figure 2C1,C2, co‐precipitation of RAGE with RIPK1 in BV2 microglia was significantly increased under high‐glucose conditions, and this increase was abolished by the RAGE antagonist FPS‐ZM1 (*F*(4, 10) = 176.30, HG vs NG, *p* < 0.001; FPS vs HG, *p* < 0.001; DMSO vs FPS *p* < 0.001; Figure 2C1,C2). Similarly, in the hippocampus of db/db mice, treatment with the RAGE inhibitor blocked the RAGE–RIPK1 interaction (*F*(3, 8) = 79.76, db/db vs db/m, *p* < 0.001; FPS vs db/db, *p* < 0.001; Figure 2D1,D2). We also used immunostaining to measure the colocalization of RAGE and RIPK1 in BV2 microglia under high‐glucose conditions. With high glucose, RAGE expression in BV2 microglia was elevated and strongly colocalized with RIPK1 (*F*(4, 45) = 109.20, HG vs NG, *p* < 0.001; Figure 2E1,E2). By contrast, cells with high glucose but treated with FPS‐ZM1 exhibited lower levels of RAGE and less RAGE–RIPK1 co‐localization (*F*(4, 45) = 109.20, FPS vs HG, *p* < 0.001; Figure 2E1,E2).

### 3.3 RAGE accelerates activation of the RIPK1‐related signaling pathway in BV2 microglia

**FIGURE 3:**
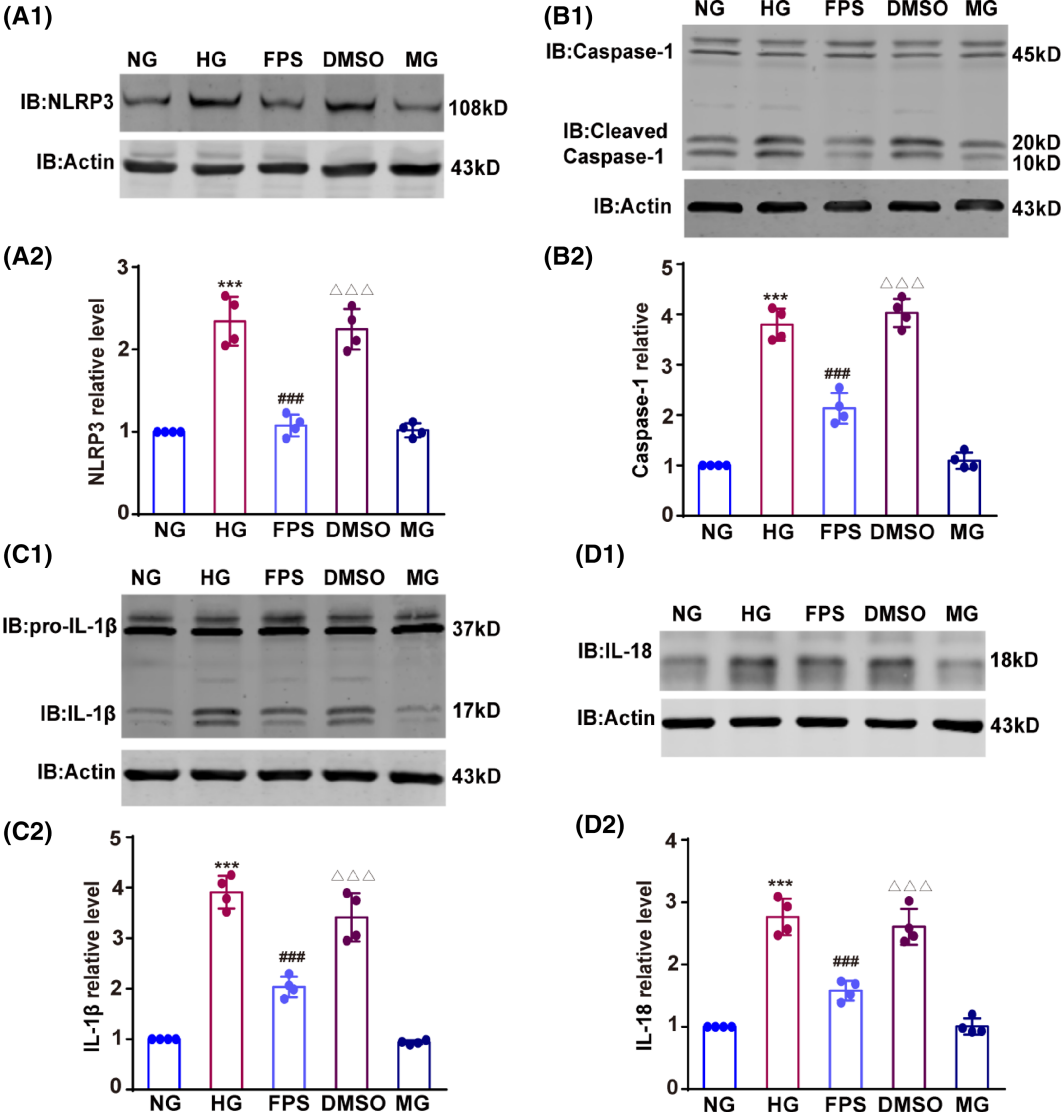
RAGE‐RIPK1 interaction increases NLRP3 inflammasome activation in a high‐glucose environment. (A1 and A2) Expression of NLRP3 in BV2 cells was measured, and the optical density is displayed as the fold change relative to the NG group. (B1 and B2) The expression of cleaved caspase‐1 was detected by Western blotting, and the relative intensity is shown as the fold change relative to the NG group. (C1–D2) The expression of IL‐1β and IL‐18 was tested with anti‐IL‐1β and anti‐IL‐18 antibodies, respectively, and the intensity is represented as the fold change relative to the NG group. Results were shown as the mean ± SD. *n* = 4 in each group. Data were analyzed by one‐way ANOVA followed by Tukey's test. ***p < 0.001 compared with the NG group, ^###^ *p* < 0.001 compared with the HG group, and ^△△△^ *p* < 0.001 compared with the FPS group.

Since RAGE alters RIPK1 phosphorylation, we further tested its role in regulating RIPK1‐mediated inflammatory signaling pathways. The NLRP3 inflammasome is a critical mediator of the RIPK1‐related inflammatory response in microglia,^21^ so first we tested whether RAGE affects NLRP3 expression. High glucose triggered an increase in NLRP3 expression in BV2 microglia, and FPS‐ZM1 significantly attenuated this increase (*F*(4, 15) = 55.27, HG vs NG, *p* < 0.001; FPS vs HG, *p* < 0.001; DMSO vs FPS, *p* < 0.001; Figure 3A1,A2). We further assessed the activity of the NLRP3 inflammasome during microglia subjected to high glucose. We found that the cleaved active form of caspase‐1, the abundance of mature IL‐1β protein, and the expression of IL‐18 were markedly increased in BV2 microglia treated with high glucose. Notably, FPS‐ZM1 was able to dramatically diminish these effects, indicating that RAGE overexpression plays a role in activating the NLRP3 inflammasome (*F*(4, 15) = 139.20 (B2), 99.33 (C2) and 68.71 (D2), HG vs NG, *p* < 0.001; FPS vs HG, *p* < 0.001; DMSO vs FPS, *p* < 0.001 in B2, C2, and D2; Figure 3B1–D2). Together, these findings demonstrate that, under high‐glucose conditions, RAGE activates RIPK1 with subsequent activation of the NLRP3 inflammasome, leading to the production of inflammatory factors in microglia.

### 3.4 RAGE directly binds RIPK1 through its intracellular 362–367 domain

**FIGURE 4:**
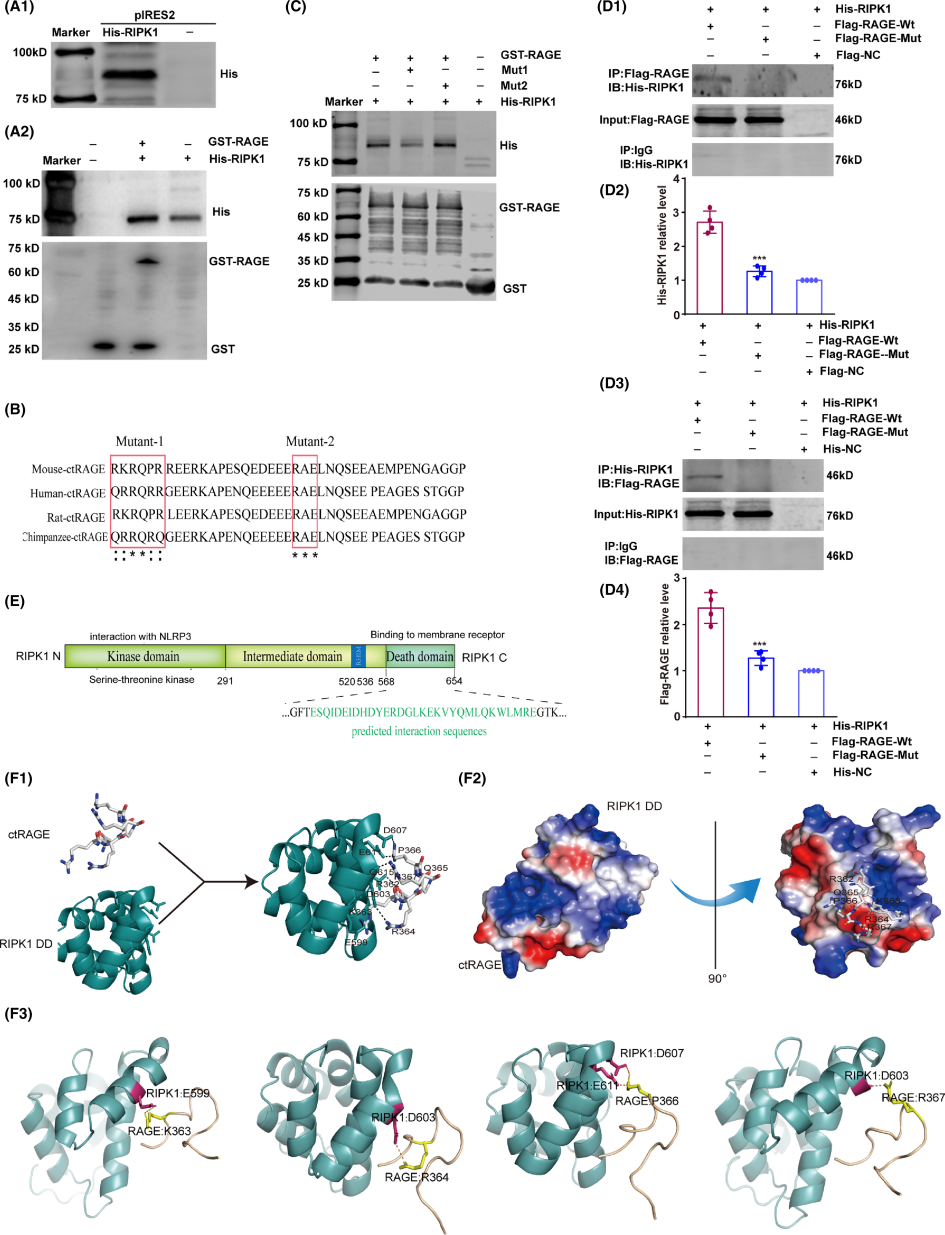
RAGE intracellular AAs 362–367 binds directly RIPK1. Mut1: Mutant1 of RAGE (AAs 362–367); Mut2: Mutant2 of RAGE (AAs 383–385). ctRAGE: C‐terminal RAGE; RIPK1 DD: The death domain of RIPK1. (A1) His‐tagged RIPK1 in pIRES2 was overexpressed in BV2 cells and verified by Western blotting with the His antibody. (A2) Direct binding of RAGE to RIPK1 was detected by Western blotting. GST‐labeled RAGE was purified and then subjected to a GST pull‐down assay. His‐tagged RIPK1 was pulled down by GST‐tagged RAGE. (B) The amino‐acid sequence of C‐terminal RAGE in mouse, human, rat, and chimpanzee. The two mutated domains in mouse RAGE (AAs 362–367 and 383–385) are outlined in red: indicates highly homologous, and *indicates completely homologous. (C) GST pull‐down assay showing that RAGE Mut1 markedly reduced the interaction between RAGE and RIPK1, but RAGE Mut2 did not. (D1 and D2) Wild‐type or mutated Flag‐tagged RAGE and His‐tagged RIPK1 were co‐transfected into BV2 cells. The interaction was detected by co‐immunoprecipitation followed by Western blotting; the relative level of His‐RIPK1 is displayed as the fold change relative to the His‐RIPK1 + Flag‐RAGE‐Wt group. *n* = 4 in each group. (D3 and D4) The co‐precipitation of Flag‐tagged RAGE and His‐tagged RIPK1 was assessed by co‐immunoprecipitation followed by Western blotting with Flag antibody; the optical density of Flag‐RAGE is shown as the fold change relative to the His‐RIPK1 + Flag‐RAGE‐Wt group. *n* = 4 in each group. The relative levels of Flag‐tagged RAGE and His‐tagged RIPK1 were analyzed with a one‐way ANOVA followed by Tukey's test. Data are shown as the mean ± S.D. ***p < 0.001 compared with the NG group or the His‐RIPK1 + Flag‐RAGE‐Wt group. (E) The pattern‐structure diagram for RIPK1. (F1) Cartoon linear docking figure showing the spatial details of the RAGE–RIPK1 interaction. The RKRQPR motif in C‐terminal RAGE is shown as sticks (colored by atom), and the relevant residues of RIPK1 DD are also shown as sticks. The polar‐contact bonds between RAGE and RIPK1 are indicated by dotted lines. (F2) Vacuum electrostatics map showing the spatial details of RAGE and RIPK1 binding. (F3) Binding of E599, D603, and D607 on RIPK1 DD and the RKRQPR motif. A Swiss‐model server and the Protein Data Bank database were used to analyze the spatial structure of RAGE and RIPK1. The docking model was generated by AutoDock Vina (version 1.1.2).

To further investigate whether RAGE regulates RIPK1 phosphorylation via direct binding to its regulatory regions, we tested for a direct interaction with a GST pull‐down assay. We generated recombinant His‐tagged pIRES2‐RIPK1 plasmids and transfected them into BV2 microglia and then purified the extracted proteins using nickel columns (Figure 4A1). A GST‐pGEX‐4T‐1‐RAGE recombinant plasmid was also overexpressed and purified as the bait protein, and His‐RIPK1 was the prey protein. The assay results indicate that His‐RIPK1 combined with GST‐RAGE directly (Figure 4A2). RAGE mediates the effects of ligands or endogenous stimulators through its intracellular C‐terminal domain, thereby activating downstream signaling pathways that lead to cellular injury.^29^ Therefore, we next focused on clarifying the precise interacting motifs for RAGE binding to RIPK1. On the basis of the reported motifs for RAGE binding to protein and small molecule compounds, we generated two recombinant mutations in the C‐terminal of mouse RAGE (Mut1: R362A‐K363A‐R364A‐Q365A‐P366A‐R367A; Mut2: R383A‐E385A) (Figure 4B). GST pull‐down results demonstrated that the binding of RAGE and RIPK1 was diminished when RAGE was mutated at Aas 362–367, but that mutation at Aas 383–385 did not have this effect. (Figure 4C). Thus, the 362–367 AA motif in RAGE is crucial for the RAGE–RIPK1 interaction; RAGE mutated at Aas 362–367 was used in the subsequent experiments.

Next, we co‐transfected Flag‐RAGE‐Wt/Mut and His‐RIPK1 into BV2 microglia. Through co‐immunoprecipitation with anti‐Flag and anti‐His antibodies, respectively, we found that Flag‐carried RAGE‐Wt, but not RAGE‐Mut, co‐immunoprecipitated with His, suggesting that binding between RAGE and RIPK1 in BV2 microglia was prevented by the mutated C‐terminal RAGE (*F*(2, 9) = 77.87 (D2) and 45.64 (D4), His‐RIPK1 + Flag‐RAGE + Mut vs His‐RIPK1 + Flag‐RAGE + Wt, *p* < 0.001 in D2 and D4; Figure 4D1–D4). Additionally, immunofluorescence results show that co‐localization of RIPK1 with RAGE under high‐glucose conditions was lower with mutant RAGE than wild‐type RAGE (*F*(2, 21) = 44.99, His‐RIPK1 + Flag‐RAGE + Mut vs His‐RIPK1 + Flag‐RAGE + Wt, *p* < 0.001; Figure S2A1,A2). These results verify that AAs 362–367 of RAGE mediate the interaction between RAGE and RIPK1.

The death domain (DD, AAs 568–654) plays a key role in RIPK1 binding to membrane receptors (Figure 4E), so we used molecular docking to predict the spatial characteristics of the RAGE–RIPK1 interaction. Notably, the docking results show that C‐terminal RAGE AAs 362–367 and the RIPK1 DD are critical for the formation of the RAGE–RIPK1 interaction as they are involved in the polar contacts (Figure 4F1). The vacuum electrostatic map suggests that the RIPK1 DD forms a positively charged “semi‐pocket” that largely matches the spatial structure of the RKRQPR motif in the C‐terminal RAGE domain (Figure 4F2). In particularly, 559E–363 K, 603D–363R, 607D/611E–366P, and 603D–367R in the RIPK1 DD and C‐terminal RAGE are crucial binding sites for the formation of the RAGE–RIPK1 complex, as they are involved in the polar contacts (Figure 4F3). Taken together, these findings indicate that RAGE binds directly to RIPK1 through the interaction of RAGE AAs 362–367 and the RIPK1 DD.

### 3.5 Mutation of RAGE AAs 362–367 inhibits activation of the RIPK1 signaling pathway by restricting the interaction of RAGE and RIPK1

**FIGURE 5:**
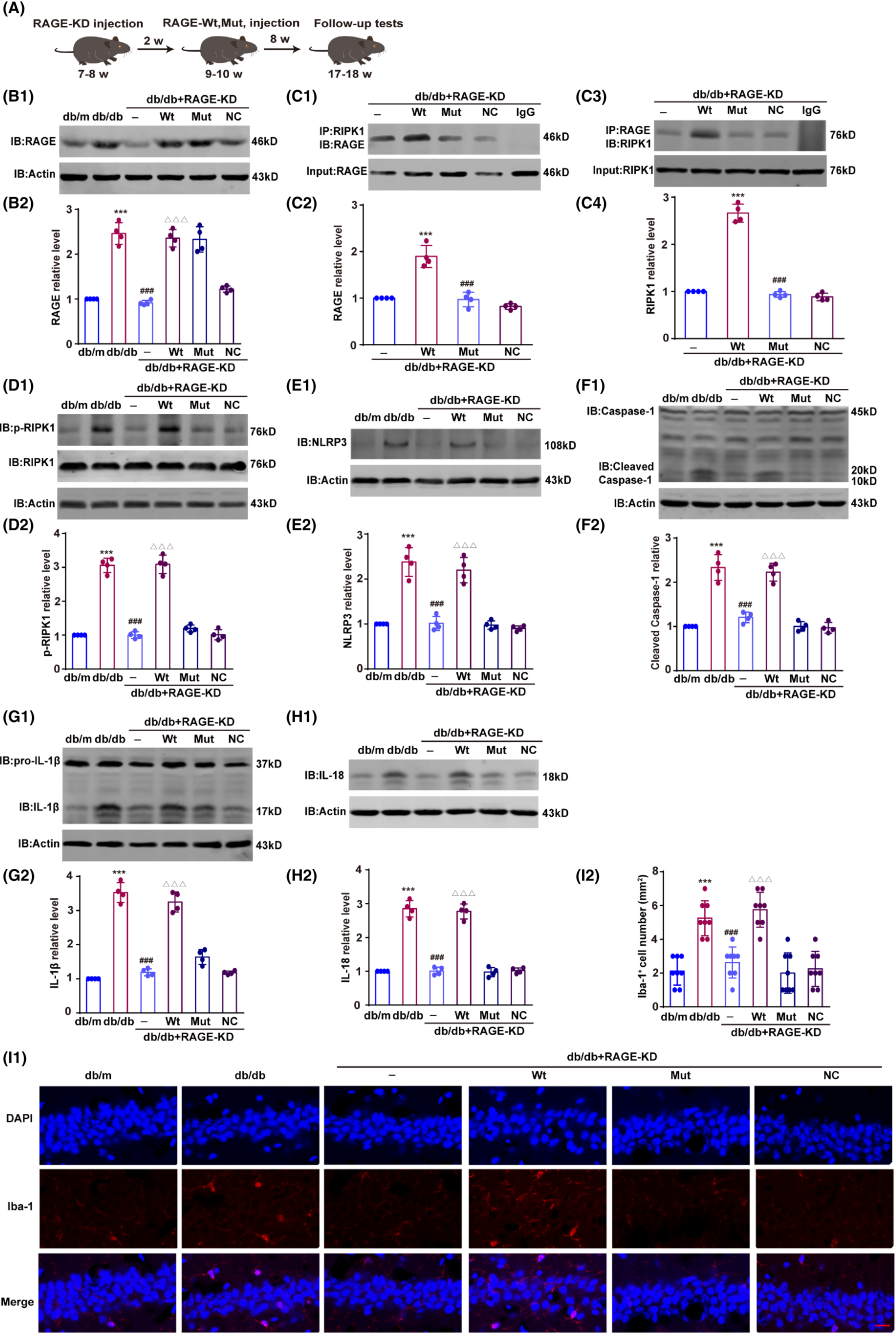
Suppression of the RAGE–RIPK1 interaction inhibits inflammation in the hippocampus of db/db mice. db/db + RAGE‐KD: db/db mice with RAGE knockdown in the hippocampus; db/db + RAGE‐KD + Wt: db/db + RAGE‐KD mice treated with wild‐type RAGE; db/db + RAGE‐KD + Mut: db/db + RAGE‐KD mice treated with mutant RAGE; db/db + RAGE‐KD + NC: db/db + RAGE‐KD mice treated with a nonsense control. (A) Schematic of the experimental diagram. (B1 and B2) Wild‐type and mutant RAGE expression in the hippocampus, with the intensity represented as the fold change to the db/m group. (C1–C4) Interaction of RAGE and RIPK1 in the hippocampus. Optical density is presented as the fold change to the db/db + RAGE‐KD group. D1‐D2: p‐RIPK1 levels detected by Western blotting; the relative intensity of the protein is displayed. (E1–H1) Expression of NLRP3, cleaved caspase‐1, IL‐1β, and IL‐18 in the hippocampus (relative intensity). (I1) Microglial activation in the hippocampus was detected by immunofluorescence with an anti‐Iba‐1 antibody. RAGE expression is labeled in red, and the cellular nuclei are labeled in blue by DAPI. Scale bar is 20 μm (400× magnification). (I2) The number of Iba‐1^+^ microglia per 1‐mm length was calculated. n = 4 in each group (two brain sections per mouse). Data were shown as the mean ± S.D and were analyzed by one‐way ANOVA followed by Tukey's test. ***p < 0.001 compared with the db/m group (B2, D2, E2, F2, G2, E2, H2, and I2) or db/db + RAGE‐KD group (C2 and C4). ^###^ *p* < 0.001 compared with the db/db group (B2, D2, E2, F2, G2, E2, H2, and I2) or db/db + RAGE‐KD + Wt group (C2 and C4), and ^△△△^ *p* < 0.001 compared with the db/db + RAGE‐KD group (B2, D2, E2, F2, G2, E2, H2, and I2).

T2DM is characterized by hyperglycemia with chronic low‐grade inflammation, leading to severe complications.^30^, ^31^ Activation of the RIPK1 signaling pathway in microglia drives neuroinflammation through mediating RIPK1‐dependent downstream proteins.^32^ Therefore, we investigated the effect of mutated RAGE on the activation of the RIPK1 signaling pathway in microglia of the hippocampus, and the experimental schedule is shown in Figure 5A. To suppress endogenous RAGE, LV‐RAGE‐shRNA was microinjected bilaterally into hippocampal CA1. Two weeks later, GFP‐tagged AAV‐RAGE‐Wt or AAV‐RAGE‐Mut under the microglial CD68 promoter was injected bilaterally into the hippocampus to overexpress mutant RAGE in hippocampal microglia. LV‐RAGE‐shRNA decreased the level of RAGE in db/db mice, and injections of wild‐type and mutated AAV‐RAGE both elevated RAGE expression in hippocampus to the same extent (*F*(5, 18) = 71.71, db/db vs db/m, *p* < 0.001; db/db + RAGE‐KD vs db/db, *p* < 0.001; db/db + RAGE‐KD + Wt vs db/db + RAGE‐KD, *p* < 0.001; db/db + RAGE‐KD + Mut vs db/db + RAGE‐KD + Wt, *p* < 0.001; Figure 5B1,B2). An immunofluorescence assay also showed that RFP‐tagged AAV‐RAGE‐Wt/Mut was overexpressed in the hippocampal CA1 region despite the early knockdown of RAGE (Figure S3A).

Consistent with the results in BV2 microglia, binding of mutated RAGE with RIPK1 in the hippocampus of db/db mice was dramatically reduced compared with the binding of wild‐type RAGE (*F*(3, 12) = 45.98 (C2) and 254.20 (C4), db/db + RAGE‐KD + Wt vs db/db + RAGE‐KD, *p* < 0.001; db/db + RAGE‐KD + Mut vs db/db + RAGE‐KD + Wt, *p* < 0.001 in C2 and C4; Figure 5C1–C4). We next analyzed the contribution of the RAGE AA 362–367 mutation to RIPK1 phosphorylation and found that hyperglycemia‐induced RIPK1 phosphorylation was dramatically lower with mutated RAGE than with wild‐type RAGE (*F*(5, 18) = 161.70, db/db + RAGE‐KD + Wt vs db/db + RAGE‐KD, *p* < 0.001; db/db + RAGE‐KD + Mut vs db/db + RAGE‐KD + Wt, *p* < 0.001; Figure 5D1,D2). Because activation of the NLRP3 inflammasome is involved in RIPK1‐mediated neuroinflammation in microglia,^20^, ^21^ we also assessed the expression of NLRP3 and its related inflammatory factors to determine whether similar mechanisms regulate the inflammatory program in the hyperglycemic environment. As expected, the levels of NLRP3, cleaved caspase‐1, IL‐1β, and IL‐18 were strongly elevated in db/db mice, but these increases were blocked by knockdown of RAGE. As anticipated, activation of the NLRP3 inflammasome in RAGE‐silenced db/db mice was reinstated by AAV‐RAGE‐Wt but not AAV‐RAGE‐Mut (F(5, 18) = 51.61 (E2), 59.98 (F2), 132.50 (G2) and 143.50 (H2), db/db + RAGE‐KD + Wt vs db/db + RAGE‐KD, *p* < 0.001; db/db + RAGE‐KD + Mut vs db/db + RAGE‐KD + Wt, *p* < 0.001 in E2, F2, G2 and H2; Figure 5E1–H2). An Iba1 immunofluorescence assay demonstrated that overexpression of wild‐type RAGE activated microglia in the hippocampus, but microglial activity remained inhibited with mutated RAGE (*F*(5, 42) = 22.42, db/db vs db/m, db/db + RAGE‐KD vs db/db, db/db + RAGE‐KD + Wt vs db/db + RAGE‐KD, *p* < 0.001; db/db + RAGE‐KD + Mut vs db/db + RAGE‐KD + Wt, *p* < 0.001; Figure 5I1,I2). Consequently, in db/db mice, mutation of RAGE (AAs 362–367) suppresses neuroinflammation by blocking the RAGE–RIPK1 interaction, ultimately reducing activation of the RIPK1 signaling pathway and decreasing activated microglia in the hippocampus.

### 3.6 RAGE mutation ameliorates cognitive impairments in db/db mice

**FIGURE 6:**
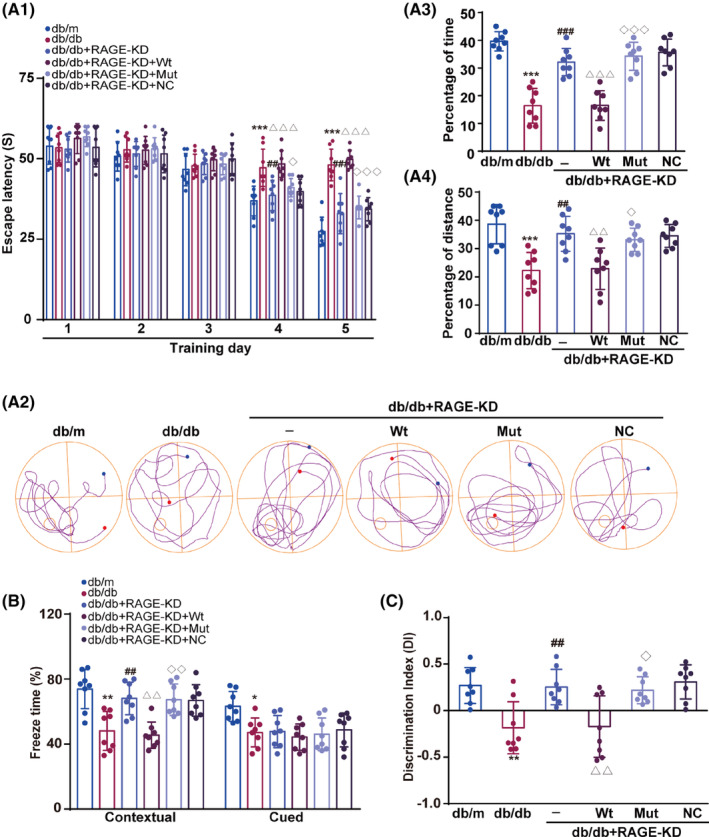
RAGE mutation ameliorates cognitive impairment in db/db mice. (A1) Escape latency of mice to reach the hidden platform on five consecutive training days. Results were analyzed by two‐way repeated‐measures ANOVA followed by Sidak's test. ***p < 0.001 compared with the db/m group, ^##^ *p* < 0.01 and ^###^ *p* < 0.001 compared with the db/db group, ^△△△^ *p* < 0.001 compared with the db/db + RAGE‐KD group, ^◇^ *p* < 0.05 and ^◇◇◇^ *p* < 0.001 compared with the db/db + RAGE‐KD + Wt group. (A2) Swim paths of representative animals from each group on day 6 without the platform (probe trial). (A3 and A4) The percentage of distance and time spent in target quadrant during the probe trial. Data were presented as the mean ± SD. Results were analyzed by one‐way ANOVA followed by Tukey's test. ***p < 0.001 compared with the db/m group, ^##^ *p* < 0.01 and ^###^ *p* < 0.001 compared with the db/db group, ^△△^ *p* < 0.01 and ^△△△^ *p* < 0.001 compared with the db/db + RAGE‐KD group, ^◇^ *p* < 0.05 and ^◇◇◇^ *p* < 0.001 compared with the db/db + RAGE‐KD + Wt group. (B) The ratio of freezing time in the contextual and cued fear‐conditioning tests. Data were shown as the mean ± S.D and were analyzed by one‐way ANOVA followed by Tukey's test. *p < 0.05 and **p < 0.01 compared with the db/m group, ^##^ *p* < 0.01 compared with the db/db group, ^△△^ *p* < 0.01 compared with the db/db + RAGE‐KD group, ^◇◇^ *p* < 0.01 compared with the db/db + RAGE‐KD + Wt group. (C) Discrimination index for the novel object recognition task. Data were shown as the mean ± S.D. Results were analyzed by one‐way ANOVA followed by Tukey's test. **p < 0.01 compared with the db/m group, ^##^ *p* < 0.01 compared with the db/db group, ^△△^ *p* < 0.01 compared with the db/db + RAGE‐KD group, ^◇^ *p* < 0.05 compared with the db/db + RAGE‐KD + Wt group. *n* = 8 in each group.

Finally, we investigated the effect of specific RAGE mutation on cognitive impairment in db/db mice using the Morris water maze (MWM), fear‐conditioning test, and novel object recognition (NOR) paradigms. During the first 3 days of the MWM, there were no significant differences in escape latency among the six groups. However, on the fourth and fifth days, the escape latency of db/db mice was significantly prolonged relative to db/m mice and db/db mice with knockdown of RAGE. Restoring RAGE in db/db knockdown mice with AAV‐RAGE‐Wt restored the phenotype of prolonged escape latency, but mutant RAGE did not (On the fourth day, *q*(8, 120) = 6.63 (db/db vs db/m, *p* < 0.001), *q*(8, 120) = 5.51 (db/db + RAGE‐KD vs db/db, *p =* 0.002), *q*(8, 120) = 6.27 (db/db + RAGE‐KD + Wt vs db/db + RAGE‐KD, *p* < 0.001), *q*(8, 120) = 4.74 (db/db + RAGE‐KD + Mut vs db/db + RAGE‐KD + Wt, *p =* 0.02; On the fifth day, *q*(8, 120) = 13.30 (db/db vs db/m, *p* < 0.001), *q*(8, 120) = 9.72 (db/db + RAGE‐KD vs db/db, *p <* 0.001), *q*(8, 120) = 10.85 (db/db + RAGE‐KD + Wt vs db/db + RAGE‐KD, *p* < 0.001), *q*(8, 120) = 9.60 (Wt vs db/db + RAGE‐KD + Mut vs Wt vs db/db + RAGE‐KD + Wt, *p* < 0.001); Figure 6A1). In the probe trial, db/db mice and db/db knockdown mice treated with wild‐type RAGE spent significantly less time and distance in the target quadrant than the db/m mice, the RAGE knockdown db/db mice, or the RAGE knockdown db/db mice treated with mutated RAGE (for time, *F*(5, 42)= 31.89, db/db vs db/m, db/db + RAGE‐KD vs db/db, and db/db + RAGE‐KD + Mut vs db/db + RAGE‐KD + Wt, *p* < 0.001; for distance, *F*(5, 42) = 10.52, db/db vs db/m, *p* < 0.001, db/db + RAGE‐KD vs db/db, *p =* 0.002, db/db + RAGE‐KD + Mut vs db/db + RAGE‐KD + Wt, *p* = 0.02; Figure 6A2–A4).

In the fear‐conditioning test, db/db mice froze less than db/m mice in both the contextual and cued fear‐conditioning trials. Freezing time was significantly greater in db/db knockdown mice treated with mutant RAGE than mice treated with wild‐type RAGE in the contextual fear‐conditioning test (contextual test: *F*(5, 42) = 10.41, db/db vs db/m, *p =* 0.01; db/db + RAGE‐KD vs db/db, *p* = 0.001; db/db + RAGE‐KD + Wt vs db/db + RAGE‐KD, *p* = 0.005; db/db + RAGE‐KD + Mut vs db/db + RAGE‐KD + Wt, *p* = 0.003; Figure 6B). However, in the cued fear‐conditioning test, there were no significant differences in freezing between db/db mice and db/db mice receiving different treatments (cued test, *F*(5, 42) = 4.23, db/db vs db/m, *p* = 0.02; Figure 6B). In the NOR test, hyperglycemia induced cognitive decline, as indicated by a decrease in DI in db/db mice, but this decline was not present in db/db knockdown mice treated with mutant RAGE (*F*(5, 42) = 7.98, db/db vs db/m, *p* = 0.002; db/db + RAGE‐KD vs db/db, *p* = 0.006; db/db + RAGE‐KD + Wt vs db/db + RAGE‐KD, *p* = 0.008; db/db + RAGE‐KD + Mut vs db/db + RAGE‐KD + Wt, *p* = 0.02; Figure 6C). Taken together, these findings suggest that mutation of RAGE attenuates cognitive deficits in db/db mice through disruption of RAGE binding to RIPK1.

## 4 DISCUSSION

**FIGURE 7:**
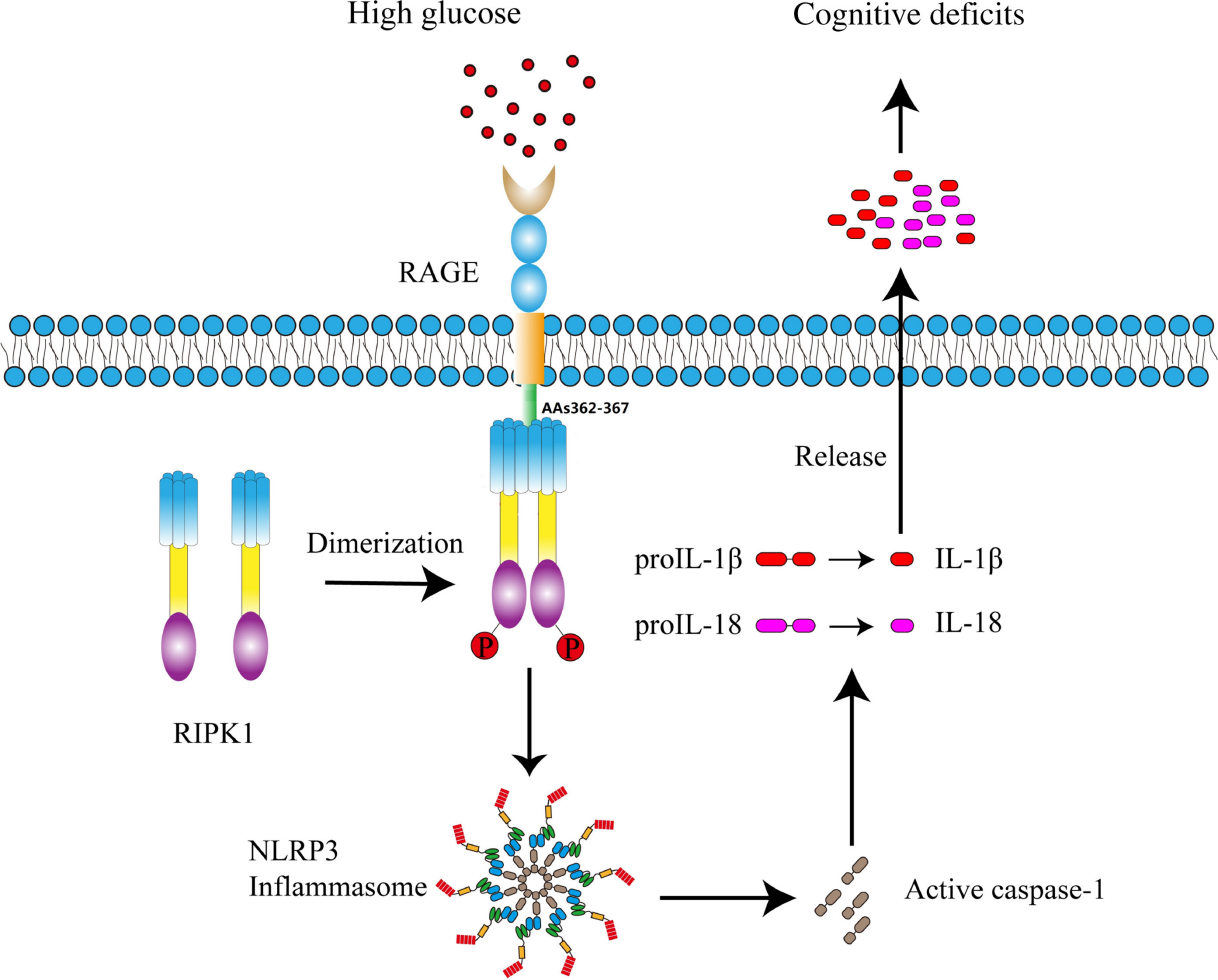
Proposed mechanism underlying RAGE regulation of cognitive deficits. Direct binding of RAGE AAs 362–367 to RIPK1 is responsible for the activation of RIPK1 and the NLRP3 inflammasome, leading to subsequent cognitive deficits.

In this study, we delineated a novel molecular mechanism by which RIPK1 is activated and its expression increased in hippocampal microglia of db/db mice. Furthermore, we showed that direct binding of RAGE AAs 362–367 to RIPK1 is responsible for activation of RIPK1 and the NLRP3 inflammasome. Furthermore, we demonstrated that mutation of RAGE AAs 362–367 is sufficient to interrupt both RAGE binding to RIPK1 and activation of the NLRP3 inflammasome by RIPK1, ultimately alleviating cognitive deficits in db/db mice (Figure 7). Our findings indicate that RAGE promotes diabetes‐induced neuroinflammation and cognitive impairment via direct binding of AAs 362–367 to RIPK1 in microglia and subsequent activation of a microglial inflammation‐related signaling pathway.

Microglia are the first line of immune defense in the CNS, capable of responding rapidly to stimuli and releasing a variety of inflammatory factors, such as tumor necrosis factor (TNF)‐α, IL‐6, and so on.^33^, ^34^ Even small changes in the CNS may stimulate microglia; at the same time, microglia may also attack normal neurons and cause them injury.^25^ T2DM is a progressive disease that causes neurodegenerative dysfunction, with the primary symptoms in the brain being a progressive loss of cognitive function and memory.^35^, ^36^ T2DM is also associated with chronic inflammation. In the brain, inflammation is closely associated with microglial activation, and long‐term inflammation eventually induces neurological damage and cognitive impairment.^37^ Nevertheless, the mechanisms by which hyperglycemia leads to microglia activation and induces inflammation remain unclear. To address this gap in knowledge, we extended previous work and found that high glucose exacerbates cytokine production as a consequence of an increase in phosphorylated (pS166) RIPK1 in microglia. Notably, one of the main mechanisms by which RIPK1 regulates neuroinflammation is activation of the NLRP3 inflammasome, which in turn promotes microglia activation.^19^, ^20^, ^21^ Therefore, activated RIPK1 appears to be an essential mediator of neuroinflammation in a high‐glucose environment and is a potential therapeutic target for cognitive disorders linked to T2DM.

A growing body of evidence, including our previous studies, has shown that RAGE is involved in the progression of cognitive impairment in diabetes.^25^ For example, T2DM patients with mild cognitive deficits have increased brain levels of RAGE.^38^ Because RAGE is an upstream modulator, RAGE‐mediated neuroinflammation requires the activation of intracellular signaling pathways. Inhibition or depletion of endogenous RAGE prevents activation of these pathways and results in decreased levels of inflammatory factors in the brain, ultimately leading to progressive improvement of cognitive function in diabetic animals.^39^, ^40^ Consistent with these studies, we found that inhibiting RAGE dampened RIPK1 phosphorylation and NLRP3 inflammasome activation in microglia and ameliorated cognitive impairments in db/db mice. Our new findings provide a plausible route for RAGE activation of RIPK1 in microglia.

Apart from the RAGE‐dependent regulatory function of RIPK1 phosphorylation, we further found that RAGE binds directly to RIPK1 via its intracellular domain at AAs 362–367. This is consistent with evidence that the intracellular portion of RAGE is the vital domain for activating downstream signaling and inducing a series of cellular processes through direct coupling with intracellular proteins. The proximal membrane motif of C‐terminal RAGE contains an unusual α‐turn that mediates the binding of RAGE to signaling proteins, including extracellular signal‐regulated kinase (ERK), mitogen‐activated protein kinase 3 (MKK3), and mammalian diaphanous‐related formin (mDia1).^25^, ^29^, ^41^ Evidence suggests that phosphorylation of RIPK1 is mediated by homologous dimerization, with the RIPK1 DD essential for dimerization and interaction with membrane receptor proteins to form complexes that lead to activation of signaling cascades.^42^ The RIPK1 DD has thus emerged as a prime mediator of cell death and transmission of inflammation‐related signals.^43^ However, the functional role of the RIPK1 DD in regulating the activation of its N‐terminal kinase domain has not been clearly elucidated. Our results suggest that, under high‐glucose conditions, RAGE binds RIPK1 directly, enhancing phosphorylation of RIPK1, and that the RAGE intracellular 362–367 domain and the RIPK1 DD may be involved in mediating this conjunction. The results from the protein docking simulation show that both AAs 362–367 of RAGE and the DD (599 E, 603 D, and 607 D) domain of RIPK1 contain hydrogen‐bond donors and acceptors, which may be essential for regulating the RAGE–RIPK1 interaction. Our study suggests that RAGE AAs 362–367 is the key motif for RAGE binding to RIPK1. In agreement with our observation, it has been reported that C‐terminal RAGE without the last 18 residues is the key motif for ligand‐induced RAGE mediation of signaling pathways.^44^

Protein–protein interactions are a key route by which proteins perform their functions: protein domains can interact physically and chemically with each other to perform necessary functions. However, abnormal protein–protein interactions are implicated in the pathogenesis of many human diseases including cancer, bacterial infection, leukemia, and neurodegenerative disease.^45^ Therefore, understanding and targeting protein–protein interactions are meaningful for the discovery of disease mechanisms and development of targeted drugs to treat disease. Small molecules that impede abnormal protein binding in disease states can have a therapeutic effect.^46^ This is supported by our finding that mutation of RAGE AAs 362–367 prevents the interaction between RAGE and RIPK1, blocking activation of RIPK1 and its downstream inflammation‐related signaling pathway in db/db mice. This is an important complement to our previous findings, which reported that the binding of RAGE to MKK3 is an essential event for activation of the p38MAPK signaling pathway in neurons under high‐glucose conditions.^25^ In the present study, we found that the interaction between RAGE and RIPK1 induces RIPK1 phosphorylation and NLRP3 inflammasome activation in microglia. These two different mechanisms by which RAGE modulates cognitive deficits may be attributed to its expression in different cell types in the brain (i.e., neurons vs microglia). Taken together, these results indicate that RAGE plays an exacerbating role in the pathogenesis of cognitive disorders in diabetes through both mechanisms, as microglial activation and neuronal damage occur almost simultaneously. These findings are consistent with the idea that transmembrane receptors such as RAGE may interact directly with various proteins in different cells to induce diverse disease processes.^47^ It is worth noting that the specific domain through which RIPK1 interacts with RAGE requires further clarification; this should be examined in a future study.

In diabetic humans and animals, impairments in spatial learning, memory, and cognition have been associated with marked changes in the hippocampus, a key brain region for many forms of learning and memory that is also sensitive to changes in glucose homeostasis.^48^ It therefore seems likely that cognitive deficits in diabetes originate from neurophysiological and structural changes in the hippocampus.^48^, ^49^ Evidence suggests that neuroinflammation in the hippocampus contributes to the pathogenesis of diabetes‐induced cognitive impairments.^50^, ^51^, ^52^ The activation of microglia plays an important role in neuroinflammation, first in the hippocampus and then in other brain areas. Neuroinflammation can lead to the breakdown of cellular and molecular processes in the hippocampus, which in turn can induce precipitous short‐term or long‐term memory deficits. It has been suggested that interventions to decrease microglial activity and neuroinflammation in the hippocampus may ameliorate cognitive dysfunction in many neurodegenerative diseases.^53^, ^54^ Adding to this, we here report that mutating RAGE attenuates microglial activity and expression of pro‐inflammatory cytokines in the hippocampus and prevents hyperglycemia‐induced cognitive decline in diabetic mice.

Previous studies have shown that diabetic patients have suffered dysmetabolism of ribose as well as glucose, indicating the potential clinical importance of ribose in diabetes and its various complications.^55^, ^56^ Both glucose and ribose participate in numerous biochemical processes, and ribose plays an active role in the glycation of protein, producing advanced glycation end products (AGEs).^56^, ^57^ In a hyperglycemic environment, this accumulation of AGEs potentially contributes to diabetes and its complications by accelerating the expression of the AGEs‐specific receptor, RAGE, and binding to it directly.^55^, ^56^ AGEs probably do not trigger neuronopathies, neuroinflammation, and cognitive dysfunction directly, but via intracellular downstream signaling pathways activated by the direct interaction of AGEs with RAGE.^25^, ^58^ Our study suggests that blocking the interaction between RAGE and RIPK1 significantly downregulates the intracellular inflammatory signaling pathway, ultimately alleviating cognitive deficits in db/db mice. Regardless, db/db mice may also experience accumulation of AGEs in the brain derived from glucose and ribose.

## 5 CONCLUSION

Our results suggest that the binding of RAGE to RIPK1 via the AAs 362–367 motif is a key molecular mechanism that enhances neuroinflammation and cognitive deficits in high‐glucose conditions. This RAGE–RIPK1 interaction was blocked by mutating the intracellular segment of RAGE, resulting in decreased neuroinflammation in the hippocampus and ameliorating deficits in cognitive function in db/db mice. Thus, RAGE AAs 362–367 might be a novel target for interventions to treat diabetes/neuroinflammation‐associated cognitive deficits.

## AUTHOR CONTRIBUTIONS

XYZ, YDZ, LG, YL, HL.CYH, and YL performed the experiments. XYZ, CJY, AKH, and YJS contributed to discussion. XYZ, YDZ, CJY, AKH, and YJS designed the project, interpreted the data, and wrote the manuscript. XYZ, CJY, and YJS are the guarantors of this work. YJS has full access to all the data and takes full responsibility for the integrity of the data.

## FUNDING INFORMATION

This work was supported by grants from the National Natural Science Foundation of China (82271205), The Traditional Chinese Medicine Science and Technology Development Program of Jiangsu Province (MS2022141), Jiangsu Province graduate student innovation project (KYCX22_2889), and the Xuzhou Special Fund for promoting scientific and technological innovation (KC21062).

## Supporting information


Figure S1.
Figure S2.
Figure S3.
Table S1.

## Data Availability

All data that support the findings of this study are included in this article and are available from the corresponding author upon reasonable request.
