# Supplementary material for: Binding of RAGE and RIPK1 induces cognitive deficits in chronic hyperglycemia‐derived neuroinflammation
Source: CNS Neurosci Ther. 2023 Sep 4;30(3):e14449. doi: 10.1111/cns.14449 (PMC10916433; doi:10.1111/cns.14449)
Supplement: Supplementary file 1 — Figure S1. Figure S2. Figure S3. Table S1. [file CNS-30-e14449-s001.docx]

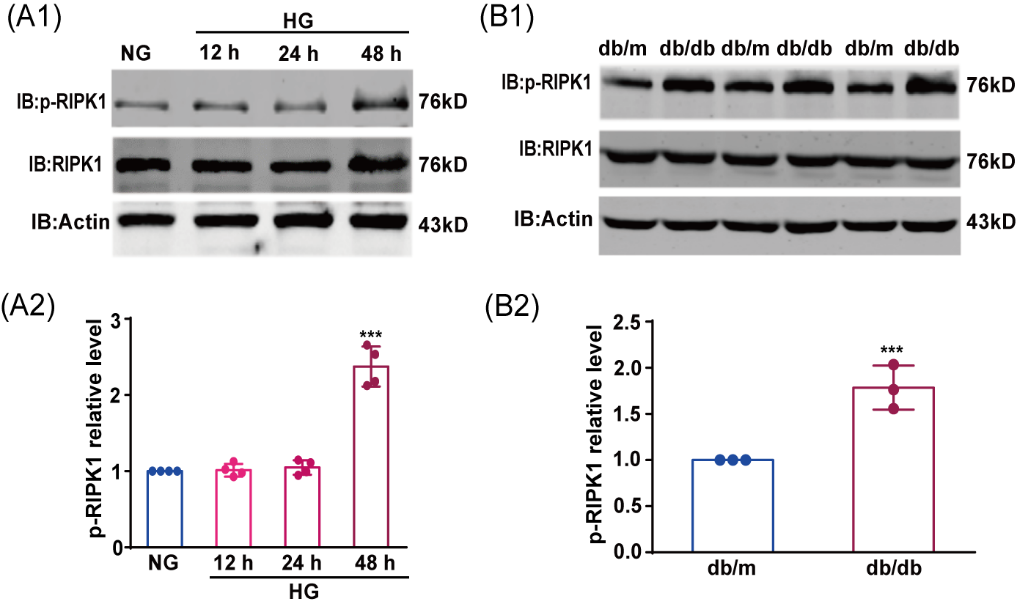


**Supplementary Figure 1:** **Increased p-RIPK1 under high-glucose conditions and in the hippocampus of db/db mice.** A1: p-RIPK1 levels in BV2 microglia were tested for different durations of high-glucose stimulation. A2: Relative level of p-RIPK1 was assessed by the optical density and presented as the fold change relative to the NG group. n = 4 in each group. Data were shown as the mean ± S.D and were analyzed by one-way ANOVA followed by Tukey’s test. *** *p* < 0.001 compared with the NG group. B1: p-RIPK1 expression in db/m and db/db mice (16–18 weeks) were detected by western blotting. B2: Fold changes in intensity relative to the db/m group. n = 3 in each group. Results were shown as the mean ± S.D. Data were analyzed by Student’s t-test. *** *p* < 0.001 compared with the db/m group.


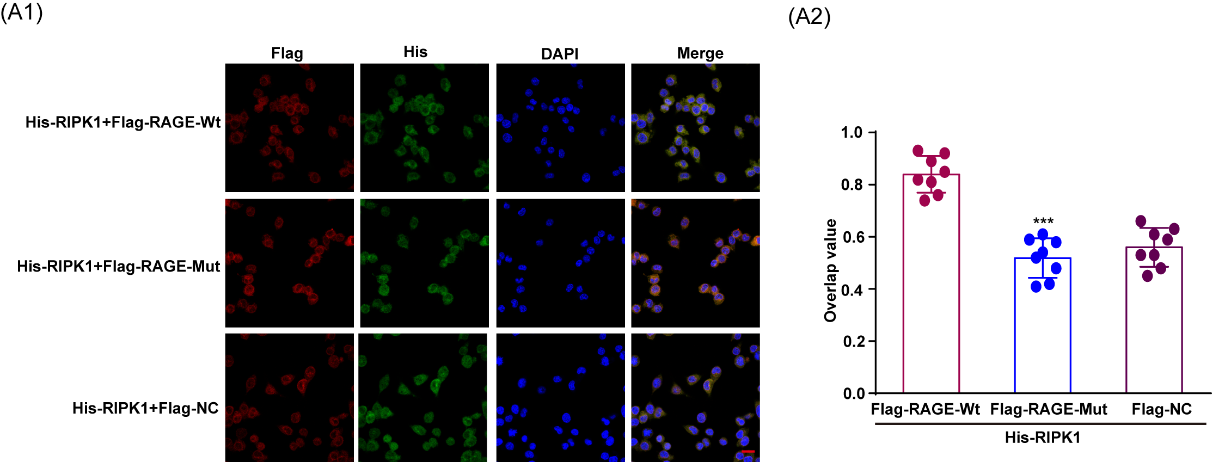


**Supplementary Figure 2: RAGE mutation at AAs 362–367 decreases co-localization of RAGE and RIPK1 in BV2 cells.** A1: His-tagged RIPK1 and wild-type or mutant Flag-tagged RAGE were co-transfected into BV2 cells. Representative laser-scanning confocal microscopy images showed the co-localization of RAGE and RIPK1. Flag-RAGE is labeled in red, His-RIPK1 is labeled in green, cellular nuclei (DAPI) are labeled in blue, and the co-localization of RAGE and RIPK1 is indicated by yellow. Scale bar = 10 µm (400× magnification). A2: Overlap values were analyzed by one-way ANOVA followed by Tukey’s test. n = 8 in each group. Data were shown as the mean ± SD. *** *p* < 0.001 compared with the Flag-RGAE-Wt + His-RIPK1 group.


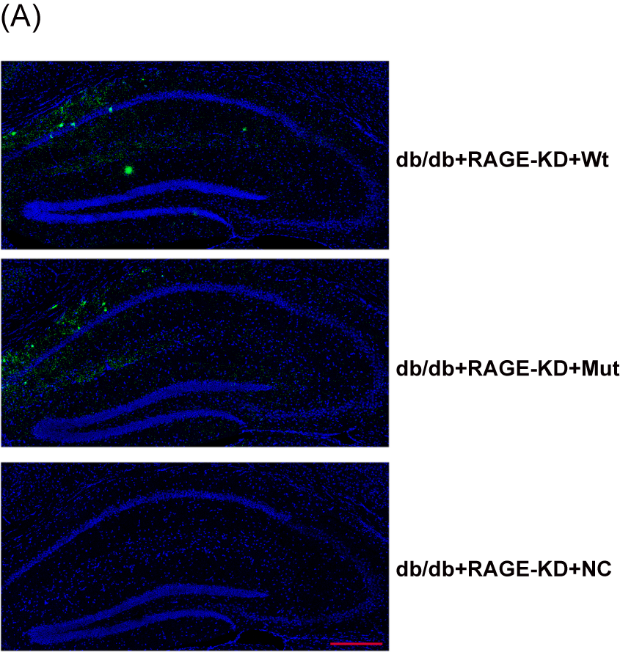


**Supplementary Figure 3: Overexpression of RFP-tagged AAV-RAGE-Wt/Mut in hippocampal sections.** A: Representative fluorescence images showed overexpression of RFP-labeled wild-type and mutant AAV-RAGE in the hippocampal CA1 subregion. Scale bar = 200 µm (100× magnification).

**Supplementary Table 1****: All antibodies, chemicals, recombinant proteins, critical commercial assays, cell lines, experimental models, oligonucleotides and recombinant DNA used in the present work.**

| Antibodies | | |
| --- | --- | --- |
| Mouse Anti-RAGE | Santa Cruz Biotechnology | sc-365154 |
| Rabbit Anti-RIPKI  Rabbit Anti-P-RIPKI | Cell Signaling Technology  Cell Signaling Technology | 3493  31122 |
| Rabbit Anti-NLRP3 | HUABIO | ET1610-93 |
| Mouse Anti-Caspase-1 | Adipogen | AG-20B-0042-C100 |
| Rabbit Anti-IL-1β | Proteintech | 16806-1-AP |
| Mouse Anti-IL-18 | Proteintech | 60070-1-Ig |
| Mouse Anti-6×His | Proteintech | 66005-1-Ig |
| Mouse Anti-Flag | Proteintech | [66008-3-Ig](http://www.ptgcn.com/products/Flag-tag-Antibody-66008-3-Ig.htm) |
| Rabbit Anti-Flag | Proteintech | [20543-1-AP](http://www.ptgcn.com/products/Flag-Tag-Antibody-20543-1-AP.htm) |
| Mouse Anti-IgG | Proteintech | B900620 |
| Rabbit Anti-IgG | Proteintech | B900610 |
| Mouse Anti-β-Actin | Cell Signaling Technology | [3700](https://www.cst-c.com.cn/products/primary-antibodies/b-actin-8h10d10-mouse-mab/3700?site-search-type=Products) |
| Rabbit Anti-GST | Proteintech | 10000-0-AP |
| Rabbit Anti-IBA1 | Wako | 019-19741 |
| Goat Anti-rabbit Alexa Fluor 488 | Proteintech | SA00013-6 |
| Goat Anti-mouse Alexa Fluor 594 | Proteintech | SA00006-3 |
| Goat Anti-rabbit Alexa Fluor 594 | Abcam | ab150080 |

| Chemicals and Recombinant Proteins | | |
| --- | --- | --- |
| FPS-ZM1 | MedChemExpress | 945714-67-0 |
| TransInTM EL Transfection Reagent | Beijing TransGen Biotech | FT201-01 |
| Protein A/G-Agarose | Santa Cruz Biotechnology | SC-2003 |
| IPTG | VICMED | 367-93-1 |
| Poly-L-Lysine | VICMED | 25988-63-0 |
| Puromycin | VICMED | 58-58-2 |
| G-418 | VICMED | 108321-42-2 |
| DMEM High Glucose | KeyGEN BioTECH | KGM12800-500 |
| [Trypsin-EDTA Solution](https://www.so.com/link?m=bfuOSXNiOCwP97XAhCgsbSmhOk+MYxZo0Um/VyYeiwS5HTpfSSffzODIGnSZhSMGrig20S/ik2/is2Hqp2et5du48gQH+11DO9ApvsF1L5CoOLIgj+bS79imKVwGsZLnG8L+wToydSA7A/QxfBlw2Tx3+7Zmae5IgUj6sO/b1jMws3JY1dciVKZYOv2Ymh0aYk6SOOwO1HAzFQ2Itz3I4dGJ73YLNXrjWM23e6Q==) | VICMED | VC2005 |
| Cryopreservation Medium | Meilunbio | MA0401 |
| QuickBlock Western | Beyotime | P0252 |
| Antibody Diluent | VICMED | VP6022 |
| Immobilon-NC Transfer Membrane | MERCK | HATF00010 |

| Critical Commercial Assays | | |
| --- | --- | --- |
| PierceTM GST Protein Interaction Pull-Down Kit | Thermo Scientific | TJ2276008 |
| Enhanced BCA Protein Assay Kit | Beyotime | P0010S |
| Plasmid microextraction Assay Kit | Tiangen Biotech | DP106 |

| Cell Lines and Experimental Models | | |
| --- | --- | --- |
| HEK-293T | Gift from Key Laboratory of brain diseases, Xuzhou Medical University |  |
| BV2 |  |  |
| Experimental Models: Organisms/Strains | | |
| Mice: db/db and db/m | Model Animal Research Center of Nanjing University |  |

| Oligonucleotides | | |
| --- | --- | --- |
| RAGE-shRNA: TGGCAAAGAAACACTCGTGAATTCAAGAGATTCACGAGTGTTTCTTTGCCATT | SANGON |  |
| NC: GTTCTCCGAACGTGTCACGTCAAGAGATTACGTGACACGTTCGGAGAATT | SANGON |  |
| Recombinant DNA | | |
| Plasmid PGEX-4T-1-GST-RAGE | SANGON |  |
| Plasmid PGEX-4T-1-GST-RAGE 362-367 (RKRQPR/AAAAAA) | SANGON |  |
| Plasmid PGEX-4T-1-GST-RAGE R383A/E385A | SANGON |  |
| Plasmid PGEX-4T-1-GST | SANGON |  |
| Plasmid pIRES2-EGFP-His-RIPK1 | SANGON |  |
| Lentivirus hU6-MCS-CMV-Puromycin-RAGE-shRNA | GeneChem |  |
| Lentivirus hU6-MCS-CMV-Puromycin-NC | GeneChem |  |
| Lentivirus Ubi-MCS-3FLAG-SV40-Neomycin-RAGE | GeneChem |  |
| Lentivirus Ubi-MCS-3FLAG-SV40-Neomycin-RAGE 362-367 (RKRQPR/AAAAAA) | GeneChem |  |
| Lentivirus Ubi-MCS-3FLAG-SV40-Neomycin-NC | GeneChem |  |
| pAAV-CD68p-MCS-EGFP-3Flag-SV40-PolyA-RAGE362-367 (RKRQPR/AAAAAA) | GeneChem |  |
| pAAV-CD68p-MCS-EGFP-3Flag-SV40-PolyA-RAGE | GeneChem |  |
| pAAV-CD68p-MCS-EGFP-3Flag-SV40 PolyA | GeneChem |  |
